# Protective effect of ellagic acid against high-glucose-induced injury in human umbilical venous endothelial cells

**DOI:** 10.22038/AJP.2023.22910

**Published:** 2024

**Authors:** Somayeh Sheikh, Hesam Dehghani, Hamid Reza Kazerani

**Affiliations:** 1 *Department of Basic Sciences, Faculty of Veterinary Medicine, Ferdowsi University of Mashhad, Mashhad, Iran*; 2 *Stem Cell Biology and Regenerative Medicine Research Group, Research Institute of Biotechnology, Ferdowsi University of Mashhad, Mashhad, Iran*

**Keywords:** Endothelial dysfunction, Ellagic acid, Adropin, Anti-oxidant, Hyperglycemia

## Abstract

**Objective:**

There is escalating evidence suggesting the beneficial effects of ellagic acid (EA) on the cardiovascular system. The aim of the present study was to investigate the protective effect of EA in human umbilical vein endothelial cells (HUVECs) against high glucose (HG)- induced endothelial dysfunction and to study the potential roles of adropin and nitric oxide (NO) in this regard.

**Materials and Methods:**

The experimental groups consisted of normal and HG (30 mM, 48 hr)-treated HUVECs incubated without or with 5 or 10 μM of EA (6 groups of at least 6 replicates, each). The cell count and viability were studied. Moreover, the markers of the redox state, including malondialdehyde (MDA), the activities of superoxide dismutase (SOD) and catalase enzymes, and ferric reducing anti-oxidant power (FRAP), were assayed. The levels of adropin and eNOS gene expression were also studied using RT-qPCR.

**Results:**

A high concentration of glucose reduced cell count and caused lipid peroxidation, reduced anti-oxidant capacity of the cells, decreased NO levels, and downregulated the expression of *NOS3* (encoding eNOS) and *ENHO* (encoding adropin) genes. Ellagic acid reversed all these effects.

**Conclusion:**

These results suggest a significant protective effect for EA against HG-induced injury in HUVECs. The improved redox state and upregulation of *NOS3* and *ENHO* genes seem to play critical roles in this regard.

## Introduction

Cardiovascular diseases (CVDs), including peripheral artery disease, coronary artery disease, myocardial infarction, and stroke, are the most important causes of mortality worldwide (Frieden et al., 2012). In 2019, there were approximately 17.9 million deaths due to CVDs, which is 31% of all-cause global deaths (World Health Organization, 2021). This annual mortality is expected to escalate to 23.6 million by 2030 (Kralj and Brkić Biloš, 2013). A variety of risk factors are involved in the morbidity and mortality of CVDs, including but not limited to obesity, hypertension, dyslipidemia, and diabetes (Giugliano et al., 2021). Type II diabetes (T2D) is the most important risk factor in this regard and accounts for two-thirds of all CVD-caused mortalities (Low Wang et al., 2016). 

Diabetes mellitus (DM) is defined by a hyperglycemic status. The occurrence of DM is increasing considerably worldwide. As predicted by the International Diabetes Federation, 592 million people worldwide are expected to have DM by 2035 (Guariguata et al., 2014; Vaidya et al., 2015). Diabetes mellitus is a metabolic disease that is recognized as an independent predisposing factor for CVDs. Diabetes mellitus is associated with microvascular manifestations such as retinopathy, nephropathy, and neuropathy, and macrovascular manifestations such as myocardial infarction, stroke, and coronary artery disease. The chance of macrovascular complications is two to four times higher in patients with T2D compared to patients without T2D (Vaidya et al., 2015).

Endothelial cell (EC) dysfunction has an important role in the pathogenesis of both diabetes and CVDs (Roberts and Porter, 2013). Endothelial damage is characterized by the incapability of endothelium to modulate vascular homeostasis, in which the physiological balances of vasoconstrictive, pro-thrombotic, and pro-inflammatory effects are impaired and the risks of atherosclerosis and coronary heart disease are increased (Xu and Zou, 2009). Accordingly, several studies have demonstrated that high levels of reactive oxygen species (ROS), decreased bioavailability of nitric oxide (NO), and modification of endothelial permeability are associated with hyperglycemia in endothelial damage (Funk et al., 2012; Li et al., 2017; Mazrouei et al., 2019). In addition, studies of diseased human coronary arteries showed that about 60% of total vascular ROS is produced by NADPH oxidase (NOX), and NOX4 has the most important role in ROS generation in HUVECs (Rozentsvit et al., 2017; Sun et al., 2016).

Nitric oxide is an important modulator involved in vascular homeostasis. In addition, it has a significant anti-atherogenic role (Farah et al., 2018). Nitric oxide has two distinct pathways to regulate cardiovascular function: stimulation of protein kinase G (PKG) by activating soluble guanylate cyclase and direct S-nitrosylation of proteins (Maron et al., 2013). Nitric oxide is produced from its precursor, L-arginine, by endothelial nitric oxide synthase (eNOS) (Mazrouei et al., 2019). 

Adropin is a 76 amino acid peptide hormone that is encoded by the energy homeostasis associated (*ENHO*) gene (Kumar et al., 2008). The kidney, pancreas, liver, brain, heart, coronary artery endothelial cells, and HUVECs express adropin (Aydin et al., 2013). Although some studies suggest adropin as a secretory hormone, it is also introduced as a membrane-bound peptide involved in cell-cell communication via the Nb-3/Notch signaling pathway in CNS (Marczuk et al., 2016; Wong et al., 2014). Three distinct receptors have been discovered for adropin, among these, the putative orphan G-protein coupled receptor, the subclass GPR19, is believed to be involved in mitochondrial cell respiration (Thapa et al., 2018). Interestingly, adropin seems to be also involved in the central regulation of water drinking via GPR19 (Stein et al., 2016). Adropin has several other functions in the central nervous system, including regulation of physical activity and motor coordination (Zhang et al., 2020). Different physiological and pathophysiological stimuli affect the levels of adropin, which is a key modulator in the homeostasis of glucose, fatty acids, and energy. It improves insulin sensitivity, and it may be involved in the pathogenesis of T2D (Zang et al., 2018). Adropin has a beneficial effect on ECs and has been recognized as a novel regulator for these cells (Topuz et al., 2013). It is a key modulator of *eNOS* gene expression and regulates the bioavailability of NO in coronary arteries (Lovren et al., 2010). 

Therapeutic approaches that prevent HG-induced oxidative stress may decrease the risk of cardiovascular-diabetic complications (Li et al., 2017). Anti-oxidant therapy is considered a new approach to defeating endothelial dysfunction by decreasing ROS generation, improving oxidative balance, and increasing the production of endothelial NO (Roberts and Porter, 2013). Recently, different anti-oxidants have been found to activate and increase the synthesis of endothelial NO (Sabando et al., 2020). 

Ellagic acid (EA) is a phenolic compound that can be found in the form of ellagitannin in fruits such as pomegranate, blackberry, strawberry, and raspberry (Larrosa et al., 2006). Several studies have demonstrated that EA can suppress oxidative stress (Baeeri et al., 2018; Ding et al., 2019; Rozentsvit et al., 2017). It is capable of exerting protective effects against oxidative stress in the aorta of diabetic mice (Rani P et al., 2013). Furthermore, many studies have shown the anti-inflammatory, anti-cancer, and anti-atherosclerotic properties of EA (Ghadimi et al., 2021; Kilic et al., 2014). In this study, we assessed the potential beneficial effect of EA on HG-induced endothelial dysfunction in HUVECs. We also investigated the possible role of some key mediators in this regard.

## Materials and Methods

**Chemicals**

Human umbilical vein endothelial cells were purchased from the Iran University of Medical Sciences (Tehran-Iran). Penicillin and streptomycin were supplied by Biowest (France). Ellagic acid, dimethyl sulfoxide (DMSO), N-(1-naphthyl) ethylenediamine dihydrochloride, sulfanilamide, glucose, and 3-[4,5-dimethylthylthiazol-2-yl]-2,5diphenyltetrazolium bromide (MTT) were purchased from Sigma (Germany). Dulbecco’s Modified Eagle Medium (DMEM) F-12, DMEM low glucose, trypsin-EDTA, and fetal bovine serum (FBS) were obtained from Gibco Life Technologies Ltd (UK). Catalase and superoxide dismutase (SOD) assay kits were obtained from ZellBio Company (Germany). Thiobarbituric acid (TBA), trichloroacetic acid, tri(2-pyridyl)-s-triazine (TPTZ), malondialdehyde (MDA), sodium acetate, hydrochloric acid (HCl), FeSO4, vanadium chloride, and FeCl3-6H2O were bought from Merck Chemical Company (Germany). Total RNA Isolation Kit was purchased from DENAzist Asia Co. (Iran). Moloney Murine Leukemia Virus (MMLV) reverse transcriptase was supplied by Thermo Fisher Scientific (USA) and 2X SYBR® qPCR master mix was provided by Amplicon (Denmark). 

**Culture of HUVECs and treatment**

**Figure 1:**
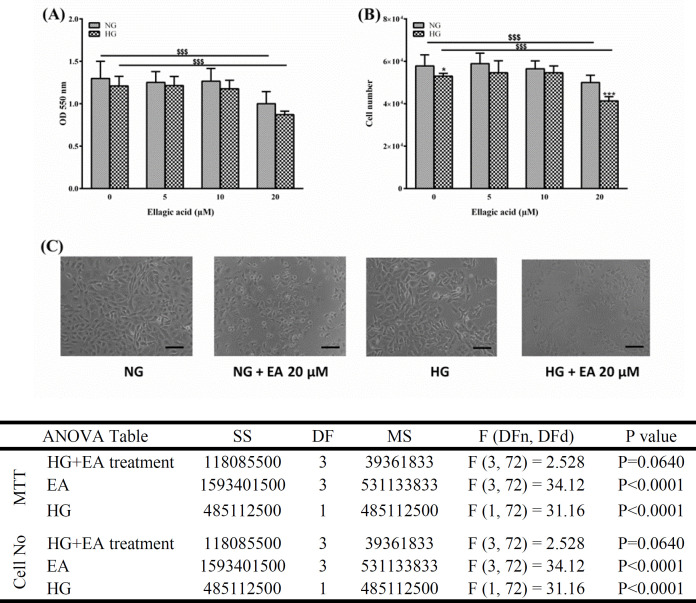
The effect of ellagic acid (EA) on MTT cell viability (A), cell count (B), and morphology (C) of human umbilical vein endothelial cells. The cells were incubated under normal glucose (NG) or high glucose (HG) concentrations for 48 hr. Data are presented as mean±SD (n=10). Scale bar = 100 μm. The asterisks indicate statistical significance compared to the adjacent normal glucose group (*p<0.05 and ***p<0.001). $$$p<0.001. SS: sum-of-squares, DF: degrees of freedom, MS: mean squares, DFn: degrees of freedom in the numerator, DFd: degrees of freedom in the denominator.

Human umbilical vein endothelial cells were cultured in DMEM F-12 mixed with penicillin (100 IU/ml), streptomycin (100 µg/ml), and FBS (10%). The cells were cultured at 37°C in an atmosphere of air (95%) and CO_2_ (5%). To avoid variations among experiments, several isogenic cell lines were derived from the original cell line and used in the following analyses. Cells had a cobblestone appearance and large dark nuclei. Upon ~70% confluency, the cells were dissociated using trypsin-EDTA. The isogenic HUVECs in different experimental groups (n = 6-10, each) were incubated using either 5.5 mM (physiological concentration) or 30 mM of glucose (high concentration), in the presence (5, 10, and 20 μM) or absence of EA for 48 hr (Baeeri et al., 2017; Baradaran Rahimi et al., 2018). To make the stock solution (10 mM), EA was dissolved in DMSO. The control groups received a similar concentration of DMSO as the vehicle. It is noteworthy that EA at 20 μM was toxic to the HUVECs as assessed by cell counting and viability tests (Figure 1); therefore, 10 μM of EA was used for the rest of the studies.

**Cell viability (MTT assay) and cell counting**

The percentage of cell viability of HUVECs in response to different concentrations of EA was measured by MTT assay. The assay is based on the reduction of MTT to formazan by mitochondrial dehydrogenases, leading to the formation of purple formazan crystals, which can be dissolved in DMSO, and its optical density can be measured (Tolosa et al., 2015). To perform the MTT assay, the culture media of wells in different experimental groups were replaced with 90 μl of a fresh one supplemented with 10 μl of MTT solution (5 mg/mL). The cells were then incubated at 37°C for 3 hr. Then, the media were discarded and replaced with 100 μl of DMSO, the plates were placed on a shaker for 30 sec, and after 10 min of incubation, the optical absorbance was measured at 550 nm using a microplate reader (Bio Tek, EPOC2, USA). To calculate the percentage of viable cells, the measured absorbance was calculated in relation to the absorbance of control cells. To calculate the number of viable cells, the HUVECs were stained with trypan blue following trypsinization and then counted using a hemocytometer under a light microscope (Inverted microscope, Nikon Instruments Inc., USA).

**Determination of endothelial nitric-oxide production**

The level of NO released from endothelial cells is a key regulator of blood vessel function. Nitric oxide has an extremely short half-life and quickly converts to nitrite and nitrate metabolites (Farah et al., 2018). To assess the level of NO, nitrate was initially reduced to nitrite using vanadium chloride. The nitrite level was then assayed using the Griess method (Egan et al., 2002). Briefly, 50 μl of sample and nitrite standards were mixed with 50 μl of freshly prepared vanadium chloride solution (200 mg in 25 ml of 1 M HCl), and then with 50 μl of Griess reagent containing N-(1-naphthyl) ethylenediamine dihydrochloride (1%) in deionized water and sulfanilamide (2%) in HCl (1.47 M). After 30 min of incubation at 37°C, the optical density was determined using a microplate reader (EPOC 2) at 550 nm. Sodium nitrite was used as a standard.

**Lipid peroxidation assay**

Accumulation of ROS, which is caused by HG-induced oxidative damage in the cells, can lead to lipid peroxidation (Oguntibeju, 2019). Lipid peroxidation was evaluated spectrophotometrically by measuring MDA in cell lysate based on Latha and Pari protocol (Latha and Pari, 2003). Briefly, samples (100 μl) were mixed with 2 ml of TBA reactive substances (TBARS) solution (containing equal volumes of 15% trichloroacetic acid, 0.25 M HCl, and 37% TBA) before being incubated at 96°C for 30 min. The samples were left to cool to room temperature (maintained at 20-22°C), before being centrifuged at 10000 g for 10 min. The absorbance of supernatants was detected at 532 nm. The amount of lipid peroxidation is expressed as equivalent nanomoles of MDA.

**Anti-oxidant enzyme activity**

HUVECs were scraped off the wells and harvested for the detection of anti-oxidant enzyme activity. Following 30 sec of sonication in cold PBS buffer, the cells were centrifuged at 15,000 g (15 min, 4°C). The supernatant was separated and used for anti-oxidant enzyme assays.

The activities of catalase and SOD enzymes were determined using commercial assay kits following the protocols of the manufacturer.

**Ferric-reducing anti-oxidant power (FRAP) assay**

The ferric-reducing anti-oxidant power (FRAP) assay was employed to verify the *in vitro* anti-oxidant capacity of EA (Benzie and Strain, 1996). Here, 10 μl of the cell lysate was supplemented with 180 μl of the fresh FRAP reagent containing FeCl_3_ (5 ml, 20 mM), sodium acetate (50 ml, 300 mM, pH 3.6), and 2,4,6-tripyridyl-s-triazine (5 ml, 10 mM). FeSO4 solution was used for plotting the standard curve. The optical density was detected at 593 nm using a microplate reader (EPOCH 2, BioTek Instruments Inc., USA) after 40 min of heating at 40°C. The FRAP values of samples were estimated using the standard curve of FeSO4 and reported as μM of FeII equivalents.

**Reverse transcription-quantitative polymerase chain reaction (RT qPCR)**

Total RNA from HUVECs in all experimental groups was isolated using the Total RNA Isolation Kit. The extracted RNA samples had an absorbance ratio of 1.8 to 2.0 at 260/280 nm. The cDNA was synthesized from 5 μg of total RNA using random hexamer primers and MMLV reverse transcriptase. The qPCR was performed in a Rotor-Gene Q real-time thermocycler (Qiagen, USA) to quantify transcripts of *ENHO* (NM_198573) and *NOS3* (NM_000603), and *ACTB* (NM_001101) genes. The reaction mixture contained 10 pmol of each primer, 2 μl template cDNA, and 10 μL of 2X SYBR® qPCR master mix. The amplification program included the following steps: 95°C for 15 min, followed by 45 cycles of 95°C for 30 sec, 30 sec annealing at 68°C for *ENHO*, 66.2°C for *NOS3*, and 60°C for *ACTB*, and extension at 72°C for 30 sec. Emission from each sample was recorded during thermal cycling, and the threshold cycle (Ct) value was calculated from raw fluorescence data using Rotor-Gene Q software. The following primer sequences were used for qPCR: *ENHO* forward, 5'-caggctcccaagccttagtcg-3' and reverse, 5'- gtggagatgtctacctgcagtc-3', *NOS3* forward, 5'- ggatgagtatgacgtggtgtcc-3' and reverse, 5'- agatgctgttgaagcggatctta-3'; *ACTB* forward, 5'-tgcagaaggagatcactg-3' and reverse, 5'-cttgctgatccacatctg-3'. The copy number for each target gene was calculated based on the relevant standard curves and was normalized against the copy number of the reference gene (*ACTB*).

**Statistical analysis**

Data are presented as means±SD. Statistical analyses and drawings of the graphs were performed and prepared using GraphPad Prism version 9 (GraphPad Software, USA). All data were tested for gaussian distribution using Kolmogorov-Smirnov test, and accordingly, parametric statistical tests were employed. Inter-group comparisons were made using a two-way analysis of variance (ANOVA) followed by Bonferroni/Dunnett tests. Differences with p-values less than 0.05 were considered statistically significant.

## Results

**Ellagic acid (EA) at low concentrations did not affect cell count or viability**

Initially, we determined the effect of EA on cell count and viability of HUVECs under both normal and HG conditions. As assessed by the MTT assay, cell viability was not altered by EA at 0-10 𝜇M following 48 hr of incubation, regardless of glucose concentration. However, EA at 20 𝜇M decreased the cell viability in both normal (5.5 mM) and HG (30 mM) groups (p<0.001) (Figure 1A). On the other hand, 30 mM glucose and 20 𝜇M EA significantly reduced the cell count compared with the control groups (p<0.001) (Figure 1B and 1C). These results indicated that cell count and viability remained stable at low EA concentrations (5, 10 𝜇M) (Figure 1). Therefore, we chose 5 and 10 µM concentrations of EA for the rest of the study.

**Ellagic acid increased NO levels in endothelial cells exposed to HG condition**

**Figure 2:**
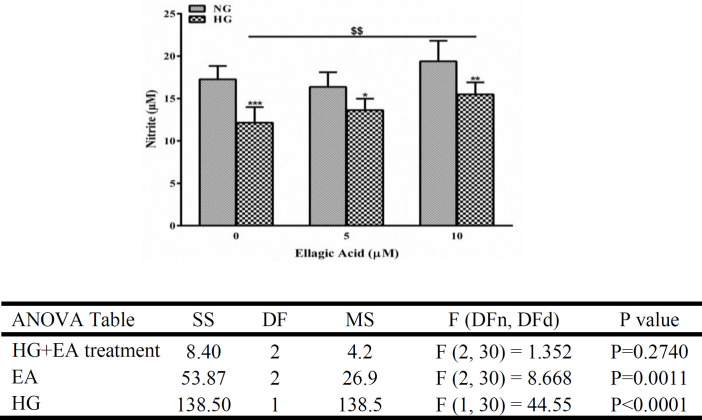
The effect of ellagic acid (EA) on the nitrite level of human umbilical vein endothelial cells. The cells were cultured using normal glucose (NG) or high glucose (HG) concentrations for 48 hr. Data are shown as mean±SD (n=6). The asterisks indicate statistical differences compared to the bordering normal glucose group (*p<0.05, **p<0.01, and ***p<0.001). $$p<0.01. SS: sum-of-squares, DF: degrees of freedom, MS: mean squares, DFn: degrees of freedom in the numerator, DFd: degrees of freedom in the denominator.

To investigate atherosclerosis in diabetic patients, the measurement of endothelial production of NO is a critical factor (Sena et al., 2013). To determine whether HG treatment decreases NO levels in ECs, we measured nitrite levels in the supernatant of cultured cells by the Griess method. As shown in Figure 2, the HG medium decreased the levels of nitrite (a metabolite of NO) compared with the control groups (p<0.001, p<0.05, and p<0.01 for 0, 5, and 10 μM of EA, respectively). However, this alteration induced by HG was reversed by 10 μM of EA (p<0.01). 

**Ellagic acid improved redox status in HG-treated HUVECs**

Oxidative stress plays a critical role in cellular injury due to hyperglycemia. Oxidative stress can induce lipid peroxidation and disrupt endogenous anti-oxidants in diabetes (Oguntibeju, 2019). The effect of EA on lipid peroxidation of HUVECs was studied by measuring MDA levels. 

**Figure 3:**
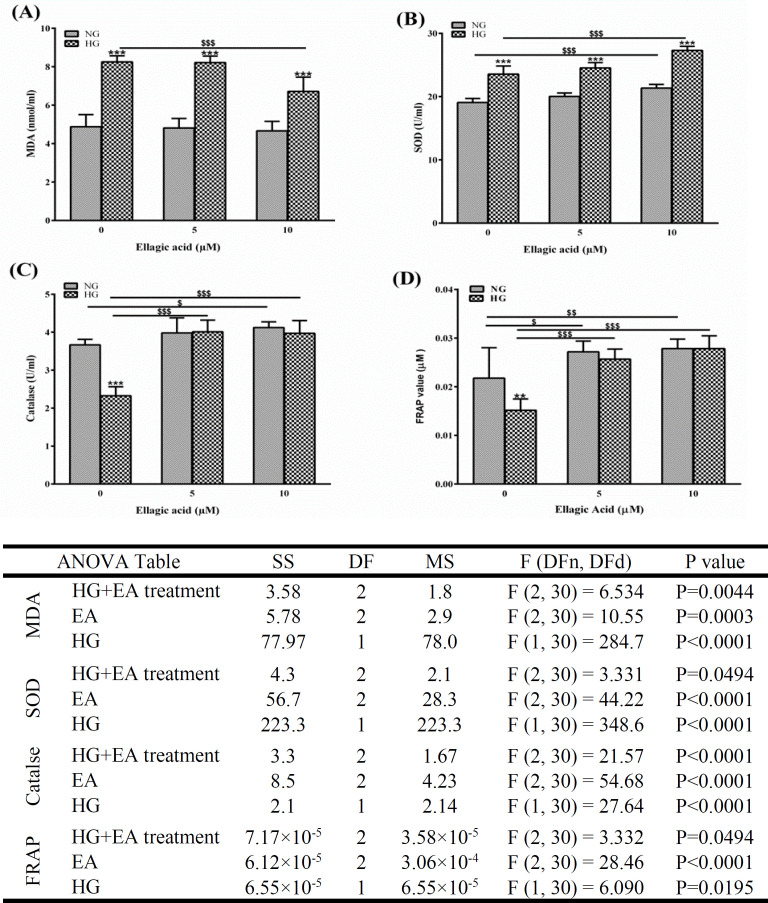
The effect of ellagic acid (EA) on redox status of human umbilical vein endothelial cells. The cells were exposed to normal glucose (NG) or high glucose (HG) concentrations for 48 hr. A: the level of malondialdehyde (MDA) as a biomarker of lipid peroxidation, B: superoxide dismutase (SOD) activity, C: catalase activity, D: ferric reducing anti-oxidant power (FRAP). Data are presented as mean±SD (n=6). The asterisks show significant differences compared to the adjacent normal glucose group (**p<0.01 and ***p<0.001). $p<0.05, $$p<0.01, and $$$p<0.001. SS: sum-of-squares, DF: degrees of freedom, MS: mean squares, DFn: degrees of freedom in the numerator, DFd: degrees of freedom in the denominator.

As shown in Figure 3A, there was a significant increase in MDA levels in HUVECs treated with a high concentration of glucose (p<0.001). This effect was significantly inhibited by EA at 10 𝜇M (p<0.001). The activity of SOD significantly increased in HG-treated groups compared with the control groups (p<0.001) (Figure 3B). Treatment with 10µM of EA caused a further increase in SOD activity. On the other hand, catalase activity in the HG treatment group was lower than that of the control group (p<0.001) (Figure 3C). The activity of this enzyme significantly increased in HUVECs which were treated with EA, regardless of glucose concentration in the medium. Consistent results were observed using FRAP assay as an indicator of the anti-oxidant capacity of cultured cells (Figure 3D). 

**Ellagic acid upregulated the expression of** ***ENHO*** **and** ***NOS3*** **genes**

**Figure 4:**
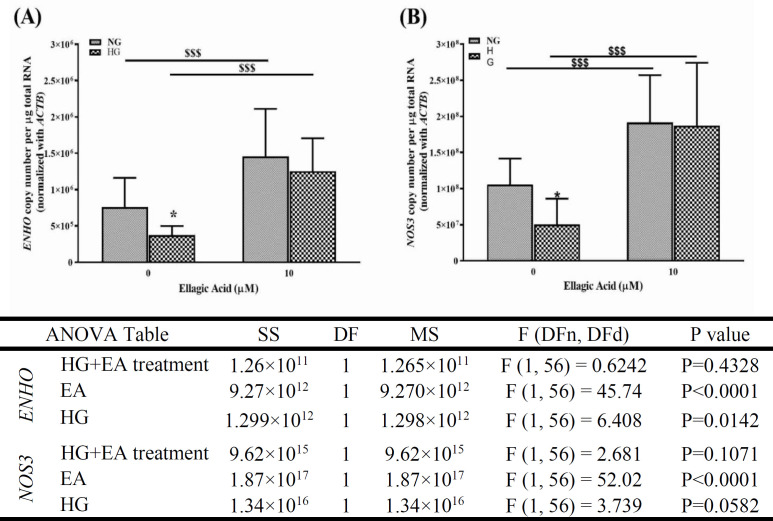
The effect of ellagic acid (EA) on the gene expression of *ENHO* (A) and *NOS3* (B). Human umbilical vein endothelial cells were incubated under normal glucose (NG) or high glucose (HG) conditions for 48 hr. Data are shown as mean±SD (n=15). The asterisks indicate significant differences with the contiguous normal glucose group (*p<0.05). $$$p<0.001. *ENHO: *energy homeostasis associated; *ACTB*: actin beta; *NOS3: *nitric oxide synthase 3. SS: sum-of-squares, DF: degrees of freedom, MS: mean squares, DFn: degrees of freedom in the numerator, DFd: degrees of freedom in the denominator.

Endothelial dysfunction is an early stage in the promotion of atherosclerosis, in which eNOS signaling is impaired (Davignon and Ganz, 2004). Therefore, we determined the level of *ENHO* and *NOS3* transcripts by RT-qPCR to identify whether endothelial dysfunction might be regulated or associated with the function of the genes involved. In HG-treated cells, the expression levels of mRNA for adropin (encoded by *ENHO* gene) (Figure 4A) and eNOS (encoded by *NOS3* gene) (Figure 4B) were significantly decreased (p<0.05). However, treatment of the cells with EA significantly increased these expression levels in both normal glucose- and HG-treated HUVECs (p<0.001). 

## Discussion

Endothelial dysfunction as an essential stage in the progression of cardiovascular implications in diabetes (Sun et al., 2016), is triggered by the dysfunction of endothelial cells (Roberts and Porter, 2013). The generation of ROS, and a reduction in the production of NO by endothelial cells, impair the function of blood vessels (Haybar et al., 2019). 

The present study demonstrated that HG levels could induce cell dysfunction through excessive cellular MDA, increased oxidative stress, and reduced NO generation. We also showed that this dysfunction can be partly explained by the downregulation of adropin and eNOS genes in HUVECs. EA prevented HG-mediated endothelial injury via scavenging ROS and upregulating adropin and eNOS genes in HUVECs.At the beginning of this research, we verified the effect of EA on the cell count and viability of HG-treated HUVECs. Although EA at 20 µM significantly decreased both parameters, they were not affected by lower concentrations of EA. 

HG concentration did not affect cell viability, but it slightly reduced the number of cells. These results were desirable since we could continue our study using lower concentrations of EA with minimal interference with cell count and viability. The endothelium plays a critical role in preserving a balance between vasoconstrictor and vasodilator agents (Farah et al., 2018). Endothelial dysfunction, characterized by impaired production of NO by ECs, is the earliest event in the progression of cardiovascular disorders, such as diabetic cardiovascular disease, hypertension, and atherosclerosis (Xu and Zou, 2009). In this research, we initially studied the effect of EA on the release of NO from HG-treated cells. We then pursued our studies to determine the possible mechanisms behind this effect. 

In this study, the amount of NO significantly diminished as a result of HG concentration, and EA restored this decline in HUVECs. Consistently, a high concentration of glucose downregulated the expression of the *eNOS* gene, and EA reversed this effect. In agreement with our results, other studies have reported a decrease in NO levels in HUVECs following 24 or 48 hr of HG treatment (Mazrouei et al., 2019; Zang et al., 2018). Similarly, both *in vitro* and *in vivo* models have revealed that eNOS expression is downregulated due to high extracellular glucose concentration (Lin et al., 2020; Mazrouei et al., 2019; Zhang et al., 2018). As far as the authors are aware, there is no report concerning the effect of EA on the NO level or *eNOS* expression in HUVECs exposed to HG concentration. However, our team has previously shown that the anti-arrhythmic and cardioprotective effects of *Punica granatum* L., the main source of EA, are mainly mediated via NO (Kazemirad and Kazerani, 2020, 2018). In another study, an EA-rich extract of *Gunnera tinctoria* increased the intracellular levels of NO in HUVECs treated with a high extracellular concentration of glucose (Sabando et al., 2020). None of these studies addressed the status of expression or eNOS activity. Interestingly, Lee et al. (2010) reported the potential clinical benefits of EA in the prevention of oxidized low-density lipoprotein (oxLDL)-associated atherogenic diseases (Lee et al., 2010). Accordingly, EA ameliorated oxLDL-impaired expression of eNOS in HUVECs via the PI3K/Akt/eNOS signaling pathway (Ou et al., 2010). Therefore, EA may be able to improve and augment PI3K/AKT/eNOS pathway, which has been reduced by hyperglycemia.

A high level of glucose is associated with excess ROS generation, which has an important role in endothelial dysfunction in diabetes (Li et al., 2017; Mazrouei et al., 2019). NADPH oxidases are the critical sources of ROS in vascular cells (Sun et al., 2016). Reactive oxygen species reduce the ability of ECs to produce NO via the inhibition of eNOS (Meza et al., 2019). In addition to the reduced formation of NO, ROS decrease the level of NO via the formation of peroxynitrite. NO may react with a superoxide anion to yield peroxynitrite. The resultant molecule is a highly reactive oxidant and nitrating agent. It leads to lipid peroxidation and damages vital biomolecules, including proteins and nucleic acid, and ends up in necrosis and apoptosis (Fleming and Busse, 2003; Su et al., 2019). In this study, excess glucose levels impaired the redox state of the cells and EA had a remarkable effect on improving the anti-oxidant capacity of the cultured cells. Consistently, the cell population and metabolism were adversely affected under HG conditions, and EA significantly reversed these effects. There is a large body of evidence regarding the overproduction of ROS in HG conditions. For instance, HUVECs incubated at 30 mM of glucose showed a significant rise in ROS generation as indicated by markers of oxidative stress (Meza et al., 2019; Yarahmadi et al., 2021; Zhou et al., 2015). Ellagic acid, a polyphenol found in some fruits and nuts, performs a variety of biological activities, including scavenging ROS (Alfei et al., 2020). In addition, it has an indirect effect on resisting oxidative stress by activating cellular anti-oxidant enzymes (Kilic et al., 2014). We did not find a similar study regarding the beneficial effects of EA on HG-treated HUVECs. However, the EA-enriched extract of *G. tinctoria* decreased cell death and the production of ROS (Sabando et al., 2020). Several studies have shown that EA, the important active compound of pomegranate, possesses anti-mutagenic, anti-inflammatory, anti-arrhythmias, anti-fibrotic, anti-cancer, anti-aging, and neuroprotective properties (Dianat et al., 2015; Sanadgol et al., 2017; Baeeri et al., 2018; Baradaran Rahimi et al., 2019; Baradaran Rahimi et al., 2020).

Consistent results have been achieved using an *in vivo* model in which diabetic patients received oral supplements of EA (Ghadimi et al., 2021). Moreover, other natural anti-oxidants have been successfully used to ameliorate the oxidative damage in HUVECs exposed to excess glucose levels. As an example, resveratrol, a potent anti-oxidant found in red grapes, has improved the oxidant and anti-oxidant balance in HG-treated HUVECs (Zhou et al., 2015). 

Adropin is an important marker of endothelial dysfunction in patients with DM (Topuz et al., 2013). It increases the production of NO through the VEGFR2-extracellular signal-regulated kinase 1/2 and VEGFR2-phosphatidylinositol 3-kinase-Akt pathways, thereby regulating the bioavailability of NO (Lovren et al., 2010). Inadequate adropin downregulates eNOS (Gao et al., 2016), which may lead to endothelial dysfunction. So, it was hypothesized that EA might increase NO via the upregulation of adropin. Our results indicate that a high level of glucose can downregulate the adropin-encoding gene, and EA upregulates this gene regardless of the levels of glucose. Although, in addition to the gene expression, the changes in nitrite levels support the modulatory effect of HG concentration and EA on the expression of adropin hormone, these findings should be further supported using protein analytical techniques, such as western blotting and/or following *ENHO* downregulation. These results should also be confirmed using *in vivo* and even *ex vivo* experiments. As far as the authors are concerned, the effects of HG condition or EA on adropin in HUVECs have not been reported in previous studies. However, clinical evidence in Chinese patients with T2D (Zang et al., 2018) and children with T1D (Polkowska et al., 2019) has shown that the serum concentrations of adropin are decreased. 

To sum up, the present study revealed that exposure to HG might cause dysfunction in HUVECs via oxidative damage and reduced NO levels. EA may be a valuable therapeutic choice to minimize cell injury by reducing oxidative stress and increasing the expression of adropin and *eNOS* genes.

This study is the first to examine the relationship between EA, the expression of *ENHO*, the level of glucose in the culture medium, and endothelial dysfunction.

One of the limitations of the current study is the absence of an examination of ROS generation. However, these effects have been reported in HG-treated endothelial cells in previous studies (Li et al., 2017; Lin et al., 2020; Meza et al., 2019). In addition, the effect of EA on the expression of *ENHO* and *NOS*_3_ genes should be examined *in vivo* using hyperglycemic animal models. The other limitation of the present study was the absence of Western blotting for the proteins involved in the signaling pathways. Our results suggest that low levels of *ENHO* expression in HG-treated HUVECs may lead to the promotion of endothelial cell dysfunction. This could also be used as a novel biomarker of endothelial dysfunction in T2D. In addition, this study provides further evidence regarding the potential prophylactic role of EA against atherosclerosis, due to its anti-oxidant properties, as well as the upregulation of *NOS3* and *ENHO* genes. Despite the cytotoxic effects at higher concentrations, EA seems to be tolerable by HUVECs at 10 μM. However, further studies are required to investigate the underlying mechanisms functionally. 

## Conflicts of interest

The authors declare that there is no conflict of interest.
